# Rotaviruses Associate with Distinct Types of Extracellular Vesicles

**DOI:** 10.3390/v12070763

**Published:** 2020-07-16

**Authors:** Pavel Iša, Arianna Pérez-Delgado, Iván R. Quevedo, Susana López, Carlos F. Arias

**Affiliations:** 1 Departamento de Genética del Desarrollo y Fisiología Molecular, Instituto de Biotecnología, Universidad Nacional Autónoma de México, Cuernavaca CP 62210, Mexico; aripd@ibt.unam.mx (A.P.-D.); susana@ibt.unam.mx (S.L.); arias@ibt.unam.mx (C.F.A.); 2 Departamento de Ingeniería Química Industrial y de Alimentos, Universidad Iberoamericana, Ciudad de México CP 01219, Mexico; ivan.quevedo@ibero.mx

**Keywords:** extracellular vesicles, rotavirus, microvesicles, exosomes

## Abstract

Rotaviruses are the leading cause of viral gastroenteritis among children under five years of age. Rotavirus cell entry has been extensively studied; however, rotavirus cell release is still poorly understood. Specifically, the mechanism by which rotaviruses leave the cell before cell lysis is not known. Previous works have found rotavirus proteins and viral particles associated with extracellular vesicles secreted by cells. These vesicles have been shown to contain markers of exosomes; however, in a recent work they presented characteristics more typical of microparticles, and they were associated with an increase in the infectivity of the virus. In this work, we purified different types of vesicles from rotavirus-infected cells. We analyzed the association of virus with these vesicles and their possible role in promotion of rotavirus infection. We confirmed a non-lytic rotavirus release from the two cell lines tested, and observed a notable stimulation of vesicle secretion following rotavirus infection. A fraction of the secreted viral particles present in the cell supernatant was protected from protease treatment, possibly through its association with membranous vesicles; the more pronounced association of the virus was with fractions corresponding to cell membrane generated microvesicles. Using electron microscopy, we found different size vesicles with particles resembling rotaviruses associated from both- the outside and the inside. The viral particles inside the vesicles were refractory to neutralization with a potent rotavirus neutralizing monoclonal antibody, and were able to infect cells even without trypsin activation. The association of rotavirus particles with extracellular vesicles suggests these might have a role in virus spread.

## 1. Introduction

Rotaviruses are non-enveloped viruses belonging to the family *Reoviridae*. Their genome is composed of 11 segments of dsRNA and codes for six structural (VP) and six nonstructural proteins (NSP) [1]. Mature rotavirus particles are formed by three concentric protein layers, known as triple layer particles (TLP), with the outermost layer being composed of two proteins, VP4 and VP7, which are responsible for the initial interactions of the virus with cell receptors. The removal of the outer layer converts TLPs into double layered particles (DLP), which are non-infectious, but transcriptionally active. The middle layer is formed by the major structural protein VP6, while the inner-most layer is formed by the VP2 protein. Inside the inner layer 11 segments of dsRNA are organized, in association with the viral polymerase (VP1) and the guanylyl-methyl-transferase (VP3) [1,2].

The early steps in the replication cycle of rotaviruses are well documented, mostly by studies carried out in MA104 cells (rhesus monkey kidney epithelium cells), with some studies having been done in other cell lines, including Caco-2 and HT-29 cells [1,3,4,5,6]. However, the latter steps in rotavirus replication, including their maturation and cell exit are not so well characterized. Rotavirus replication takes place in the cell cytoplasm, where new viral particles assemble in electrodense structures called viroplasms, which localize close to the endoplasmic reticulum (ER) [1,5]. The newly formed DLPs bud into the lumen of the ER after their interaction with NSP4, a nonstructural viral glycoprotein, resident of the ER membrane, that serves as receptor for these particles. During this process, the particles acquire a transient lipid envelope, visible by electron microscopy. Once inside the ER lumen, the enveloped particles lose the lipid bilayer and obtain the outer protein layer, composed of VP4 and VP7, by a mechanism which is poorly understood [7,8]. Recently, it was shown that the guanine nucleotide exchange factor GBF1, involved in the COPI/Arf1-mediated vesicular transport, is important for the correct post-translational processing of the viral glycoproteins VP7 and NSP4, and their assembly onto DLPs [9].

Historically, it was believed that rotaviruses are classical lytic viruses, being released from the cell by lysis [10]. However, this view changed when studies in polarized cells showed a preferential release of viral particles from the apical side, before cell lysis [3,11]. Originally, nonconventional vesicular transport, which involved small smooth vesicles in the apical face of cell, was suggested as the medium for rotavirus release [11]. Later studies suggested the involvement of specific membrane domains, called lipid rafts, during rhesus rotavirus (RRV) release from Caco-2 cells [12].

In recent years, extracellular vesicles (EV) have been associated with the release of different viruses from infected cells [13,14,15,16], and EV have been suggested to serve for dissemination of the virus particles to other cells or organs in the infected organism [17,18,19]. EV are a heterogeneous group of vesicles, produced by all cell types, which can be divided by their origin into three mayor types: exosomes (50 to 150 nm), formed from the endosomal compartment by fusion of multivesicular bodies with the cell membrane; microvesicles (from 100 nm to 1 µm), which originate from the plasma membrane by outward budding; and apoptotic bodies (from 100 nm up to 5 µm), which are produced in apoptotic cells [17,20,21].

Recently, rotavirus proteins were found to be associated with secreted vesicles in Caco-2 cells [22], and association of virions with extracellular vesicles was also observed in vitro and in vivo, and this association was reported to enhance virus infectivity and disease severity [23]. In this work, we analyze the nature of the vesicles secreted after infection of MA104 and Caco-2 cells with the rhesus rotavirus strain RRV. We found a large increase in EV secretion after rotavirus infection, and viral particles were found to sediment in association with vesicles of different sizes. Using transmission electron microscopy (TEM), the particles resembling rotavirus were found to locate externally with EV of different sizes and were also present internally in the larger size vesicles. The viral particles present inside the vesicles enter the cells without the need for protease activation of virus infectivity and were protected from action of neutralizing antibodies.

## 2. Materials and Methods

### 2.1. Cell Culture and Viral Propagation

Human colon adenocarcinoma cells (Caco-2), and rhesus monkey epithelial cell line MA104 were obtained from the American Type Culture Collection (ATCC, Manassas, VA, USA). Caco-2 cells were cultivated non-differentiated and passed every 2–3 days in Dulbecco modified essential medium (DMEM; Sigma-Aldrich, San Luis, MO, USA, Cat. D7777), supplemented with non-essential amino acids (Gibco, Billings, MT, USA Cat. 11140) and 15% fetal bovine serum (FBS; Biowest, Kansas City, MO, USA), at 37 °C and 10% CO_2_. The MA104 cells were grown in Advanced DMEM (Thermo Fisher scientific, Rockford, IL, USA) supplemented with 5% FBS, in humidified atmosphere with 5% CO_2_ at 37 °C. Antibiotics were not used in cell culture during this study. Rhesus rotavirus (RRV) strain was obtained from H.B. Greenberg (Stanford University, Stanford, CA, USA), and was propagated in MA104 cells. The virus lysates were activated prior to infection with trypsin (50 µg/mL) for 30 min at 37 °C, followed by inactivation with soybean trypsin inhibitor at same concentration (50 µg/mL).

### 2.2. Viral Infectivity Assays

The presence of infectious virus in samples was analyzed by titration in confluently grown MA104 cells in 96-well plates. Before infection, cells were washed twice with Minimum Essential Medium (MEM; Sigma-Aldrich, San Luis, MO, USA) without FBS. All samples were activated with trypsin before infection, with or without previous membrane solubilization. For titration, samples were diluted in MEM in the dilution plate and transferred to MA104 cells for 1 h at 37 °C to adsorb and enter. After this time, cells were washed to discard non-adsorbed particles, fresh medium was added and infection was left to proceed for up to 15 h. Cells were fixed with 80% acetone and infected cells were identified by immunoperoxydase assay as described previously [24].

### 2.3. Kinetics of Viral Release

To test the release of viral particles from infected MA104 and Caco-2 cells, cells were grown in 24-well plates to reach confluence. Before infection, cells were washed twice with MEM without FBS, and infected with RRV strain [multiplicity of infection (MOI) 5]. Samples were harvested from 6 h post-infection (hpi) (MA104 cells at 2-h interval) or from 12 hpi (Caco-2 cells at 3-h interval). After supernatants were harvested, equal volume of fresh medium was added to cells, to analyze cell-associated virus. All samples were activated by addition of trypsin before infection as described in Section 2.2. Infection and detection were done as described in Section 2.2. To test that cell membrane integrity was not compromised, lactic dehydrogenase (LDH) was measured in media, by in vitro toxicity assay kit, Lactic Dehydrogenase based (Sigma-Aldrich, San Luis, MO, USA), as described by the manufacturer.

### 2.4. Vesicle Purification

The cells (MA104 or Caco-2) grown in 150-cm^2^ flasks were washed twice with MEM without FBS, and infected (MOI 1) or mock treated for 1 h at 37 °C in 5 or 10% CO_2_ atmosphere. After infection, cells were washed again twice with MEM, and left in MEM without FBS, and infection was left to proceed for desired time period (10 h in MA104 cells and 18 h in Caco2 cells). After this time, supernatant was collected and different types of vesicles were purified at 4 °C by modified ExtraPEG protocol [25]. Briefly, first floating cells or very large vesicles were collected after 15 min centrifugation at 500× *g*, forming pellet 1 (P1). During following centrifugation at 2000× *g* for 30 min other large vesicles were pelleted (P2), followed by centrifugation at 9600× *g* for 20 min (P3). To the remaining supernatant, an equal volume of 16% polyethylene glycol 6000 (PEG6000) (Sigma-Aldrich, San Luis, MO, USA, Cat. 81260) and 1 M NaCl was added. Tubes were mixed and left in cold for minimum of 12 h. The small vesicles together with free viral particles were pelleted by 1 h centrifugation at 9600× *g* (PEG fraction). All pellets were resuspended in TNC solution, and washed once at their originally used conditions. After the wash, pellets were resuspended in 100 µL of TNC and stored at 4 °C for up to 10 days. When required, for some experiments, additional purification step was included. This consisted in vesicle purification using MagCapture^TM^ Exosome Isolation Kit PS (FUJIFILM Wako Pure Chemical Corporation, Osaka, Japan), following the instructions provided by the manufacturer.

### 2.5. Infectivity Associated to the EV

To measure infectious virus associated with different types of EV, these fractions were purified from supernatants of infected cells by differential centrifugation/PEG6000 precipitation as described in Section 2.4. The vesicles were added to confluently grown MA104 cells, previously washed twice with MEM, and they were left to bind and enter for 2 h at 37 °C. After cell entry, infection was left to proceed for 15 h. When desired, vesicles were treated with Triton X-100 (final concentration 0.1%) or with neutralizing monoclonal antibody 159 (nMAb159), specific for VP7 protein of rotavirus strain RRV [26]. In order to preserve cell viability, after incubation with detergent, this was removed by HiPPR^TM^ Detergent Removal Columns (Thermo Fisher scientific, Rockford, IL, USA), as described by the manufacturer.

### 2.6. Immunodetection of Cellular or Viral Proteins

To analyze the presence of specific proteins in different fractions, equal sample volume of vesicles purified from mock or virus infected cells were precipitated using the methanol/chloroform/distilled water method [27]. Precipitated proteins were separated by sodium dodecyl sulfate-polyacrylamide gel electrophoresis (SDS-PAGE) and transferred to nitrocellulose membrane (Millipore, Bedford, MA, USA). Membranes were blocked with 5% nonfat dried milk in PBS, and incubated with primary antibodies in PBS containing 0.1% milk, followed by incubation with secondary, species-specific, horseradish peroxidase-conjugated antibodies. Primary antibodies used were as follows: rabbit anti-CD63 polyclonal antibody was from Santa Cruz, (Santa Cruz, CA, USA), rabbit anti-ALIX, rabbit anti-GM130 and rabbit anti-PDI polyclonal antibodies were from Aviva System Biology, (San Diego, CA, USA), and rabbit anti-α2 integrin polyclonal antibody was from Chemicon,(Burlington, MA, USA. As secondary antibody peroxidase conjugated anti-rabbit and anti-mouse (Invitrogen, Carlsbad, CA, USA) were used. The signal was developed by Western Lightning Reagent Plus-ECL (Perkin Elmer, Waltham, MA, USA), as suggested by the manufacturer. To detect ganglioside GM1, samples (3 µL) were directly blotted onto nitrocellulose membrane and dried. Before developing, membranes were blocked in 5% nonfat dried milk, and incubated with cholera B subunit, conjugated with biotin (Sigma-Aldrich, San Luis, MO, USA), and after additional washing membranes were incubated with streptavidin peroxidase (Zymed, Thermo Fisher scientific, Rockford, IL, USA). Monoclonal antibody HS2 specific for VP5 peptide of VP4 has been described previously [28] and anti-TLP rotavirus sera were prepared in laboratory. Signal was visualized using Western Lightning as described above.

### 2.7. Nanoparticle Tracking Analysis

Nanoparticle tracking analysis (NTA) was conducted using a NanoSight NS300 (Malvern Instruments Ltd., Worcestershire, UK) to assess the hydrodynamic diameter of vesicles purified from infected and non-infected cells obtained from the differential centrifugation/PEG precipitation method. This technique uses dynamic light scattering to measure the diffusion coefficient of particles moving under Brownian motion, and converts it to the hydrodynamic diameter using the Stokes−Einstein equation [29]. To obtain the desired particle concentration, samples were diluted in PBS (1:100×). For each condition, five videos of one-minute length were recorded for each sample and analyzed using the NanoSight NTA 3.1 software [30]. Sterile and filtered PBS solutions were used as blanks for particle calculations in every measurement, and after each measurement the flushing lines were thoroughly washed to avoid contamination.

### 2.8. Transmission Electron Microscopy

Different types of vesicles obtained from infected MA104 and Caco2 cells were purified as described in Section 2.4, using the MagCapture^TM^ Exosome Isolation Kit PS. The purified fractions were fixed onto carbon vaporized coper grids and negatively stained by single-drop method with 3% solution of uranyl acetate dissolved in distilled water. Samples were afterwards observed in an EFTEM ZEISS Libra 120 electron microscope (ZEISS, CDMX, Mexico) operating at 80 KV with a GATAN Multiscan 600 HP model 794 CCD for image acquisition. Samples were analyzed using software Digitan Micrograph (Gatan, Pleasanton, CA, USA).

### 2.9. Statistical Analysis

Statistical analysis was performed with a paired T-test, using the GraphPad prism 6.0 software (GraphPad Software, Inc., San Diego, CA, USA).

## 3. Results

### 3.1. Kinetics of Rotavirus Release from MA104 and Caco2 Cells

**Figure 1:**
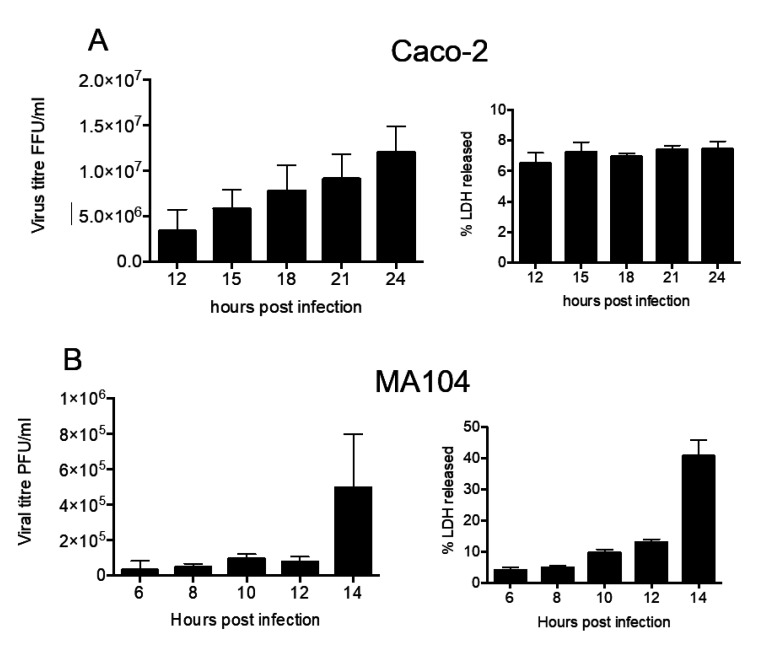
Kinetics of rotavirus release from Caco-2 and MA104 cells. Caco-2 (**A**) and MA104 (**B**) cells were infected by RRV rotavirus strain (MOI 5). At the indicated time points, the supernatant was collected and the virus present was determined as described in material and methods. In right part of the figure is the release of LDH, as a measure of cell membrane integrity. The arithmetic means and standard deviation of three independent experiments are shown.

MA104 and Caco2 cells were infected with rotavirus strain RRV (MOI of 5), and infection was left to proceed for different time periods. At the indicated times, the supernatant was collected and the presence of infectious virus was determined by titration in MA104 cells. In both cell lines, infectious virus particles were found in the cell medium starting at the earliest time point analyzed (6 h in MA104 cells and 12 h in Caco-2 cells) (Figure 1). The amount of virus in the cell culture supernatant increased as time progressed, until the maximum was obtained at 14 h in MA104 cells and at 24 h in Caco-2 cells. Of relevance, the membrane permeability of Caco-2 cells was not visibly affected during the course of experiment (Figure 1A), while MA104 cells showed cell membrane damage at 14 h post infection (hpi), as determined by the release of LDH (Figure 1B). Based on these results, to collect the virus in supernatant with the least cell membrane damage, most of following experiments were done using Caco-2 cells at 18 hpi, while experiments carried out in MA104 cells were analyzed at 10 hpi. These results confirm previous reports that found viruses in the cell medium before the cell lysis occurs [3,11,31].

### 3.2. Rotavirus Infection Increases Vesicle Secretion from Caco-2 Cells

**Figure 2:**
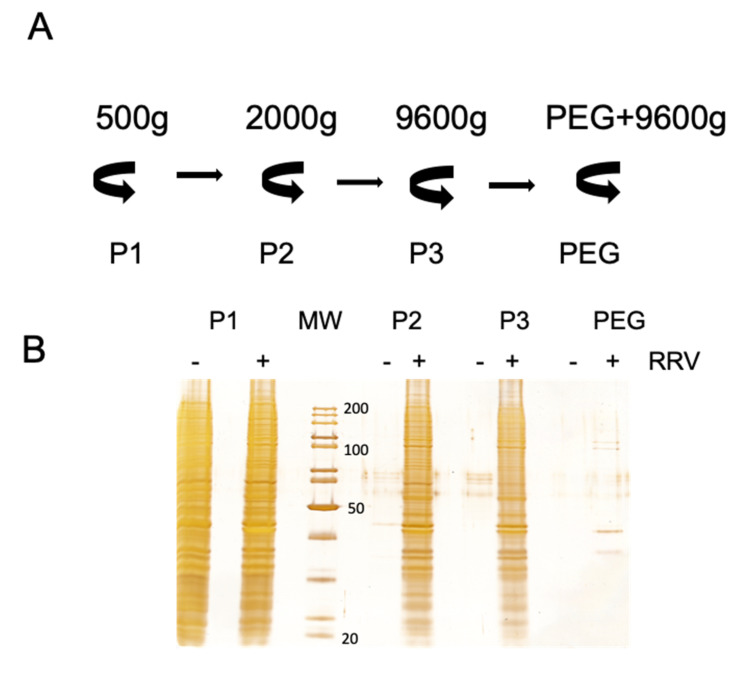

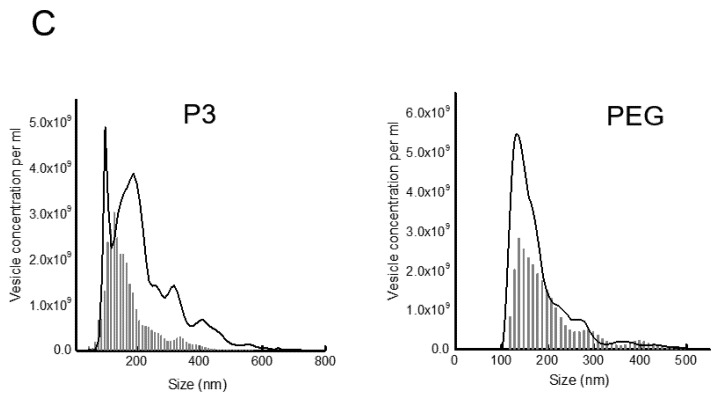
Rotavirus infection increases the secretion of extracellular vesicles in Caco-2 cells. (**A**). Schematic description of the differential centrifugation protocol used. Supernatants collected from Caco-2 cells infected with rotavirus strain RRV were centrifuged at different speed, to separate distinct size vesicles. At 500× *g*, pellet 1 (P1) was obtained; at 2000× *g*, pellet 2 (P2); at 9600× *g*, pellet 3 (P3); and after PEG 6000 incubation and centrifugation at 9600× *g*, the fraction labeled as PEG was obtained. (**B**). Caco-2 cells were infected or mock-infected with strain RRV (MOI of 1) and the supernatants were collected and processed by differential centrifugation as described in A. The same volume of each fraction from infected and mock-infected cells were separated by PAGE, and proteins were detected by silver staining. The apparent molecular weight of some marker proteins (MW) is shown. (**C**) The P3 and PEG fractions were resuspended in PBS for nanoparticle tracking analysis in the NanoSight NS300. In each experiment, five videos were recorded and used for analyses. The average of four independent experiments is shown. The black bars represent vesicles purified from mock-infected cells; grey line represents vesicles purified from infected cells. In the case of PEG fraction, due to size of free viral particles (approximately 100 nm), all particles bellow 110 nm have been removed to avoid their detection.

Secretion of extracellular vesicles is an intrinsic characteristic of all cells. The quantity and vesicle type and content depend on the physiologic conditions of the cells, and they can be affected by viral infection. To analyze the effect of virus infection on the secretion of EV in Caco-2 cells, EV were purified from infected and non-infected cells by differential centrifugation/PEG6000 precipitation (Figure 2A), and equal portions of the precipitated samples were analyzed by silver staining after SDS-PAGE separation (Figure 2B). It is clear that rotavirus infection stimulates vesicle secretion, as the protein content increased dramatically after infection in all the fractions analyzed (Figure 2B). Only in the low-speed pellet 1 the protein content obtained from mock and virus-infected cells was comparable. This fraction probably contains whole detached cells or very large vesicles, which could be a result of prolonged incubation without FBS. Similar observations were done after analysis of vesicles purified from infected MA104 cells [32].

To analyze the increase of vesicle secretion more quantitatively, the concentration and size of the vesicles purified in pellet 3 (P3) and after PEG6000 precipitation (PEG fraction) were analyzed by nanoparticle tracking. These two fractions should contain mainly microvesicles (P3) and exosomes (PEG); however, free viral particles could also be present in the PEG fraction. In the case of fraction P3, there is a clear increase in concentration and size of purified vesicles after rotavirus infection (Figure 2C, grey line). In the case of vesicles purified by PEG6000 precipitation (PEG fraction), we had to remove vesicles below 110 nm, which in case of rotavirus infection most probably represent free viral particles. The concentration of vesicles larger than 110 nm in this fraction doubled after infection, but we did not observe a noteworthy increase in vesicle size (Figure 2C). These observations suggest that rotavirus infection stimulates EV secretion.

### 3.3. Presence of Infectious Virus and Vesicular Markers in Purified Fractions

**Figure 3:**
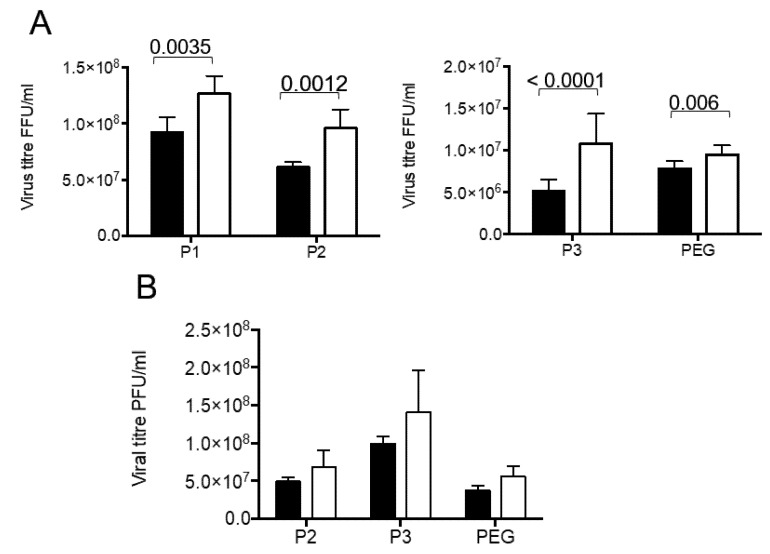
A fraction of the viral particles associated with secreted extracellular vesicles in MA104 and Caco-2 cells is protected from trypsin activation. MA104 and Caco-2 cells grown to confluence were infected with the RRV strain, for 10 or 18 h, respectively, the time at which the cell culture supernatant was collected. Different fractions were collected as described in material and methods, P1 by centrifugation at 500× *g*, P2 at 2000× *g*, P3 at 9600× *g* and PEG, by precipitation with PEG6000. All fractions were resuspended in TNC, and further processed using MEM without FBS. The infectious virus present were titrated by a standard focus forming assay, with all fractions being either treated or not with detergent (Triton X-100 0.1%) for 30 min at 37 °C before trypsin activation. (**A**) Viral titer associated with fractions purified from Caco-2 cells. (**B**) Viral titer associated with fractions purified from MA104 cells. The results are expressed as titer of infectious virus in the corresponding fraction without (full bars) or with (open bars) detergent treatment previous to trypsin activation. The results are expressed as the average and standard deviation of at least five independent experiments. Statistically significant values are shown above corresponding bars.

The fractions obtained by our differential centrifugation protocol were analyzed to detect the presence of infectious virus and diverse cellular marker molecules. Since we expected part of the virus to be present in vesicles, we compared the rotavirus infectivity in fractions with and without detergent treatment before activation with trypsin. This was done with the idea that the rotavirus particles, which were present inside the vesicles, would be protected from the trypsin action, and therefore their VP4 protein would not be cleaved into VP5 and VP8 subunits, a process needed to increase the rotavirus infectivity. Another possibility is the association of several viral particles with vesicles, such that their solubilization with detergent should liberate free infectious particles. In fractions purified from infected Caco-2 cells, an increase in virus titer was observed principally in first three pellets (P1 to P3), with little increase in virus titer in the PEG fraction (Figure 3A). Of interest, the largest increase was observed in the P3 fraction (210% ± 14%). In the case of fractions purified from infected MA104 cells, no significant increase in infectious virus was detected in neither of the three fractions tested (P2, P3 and PEG) (Figure 3B). These results show that solubilization of membranes by detergent increases infectivity of viral particles purified after infection of Caco-2 cells.

**Figure 4:**
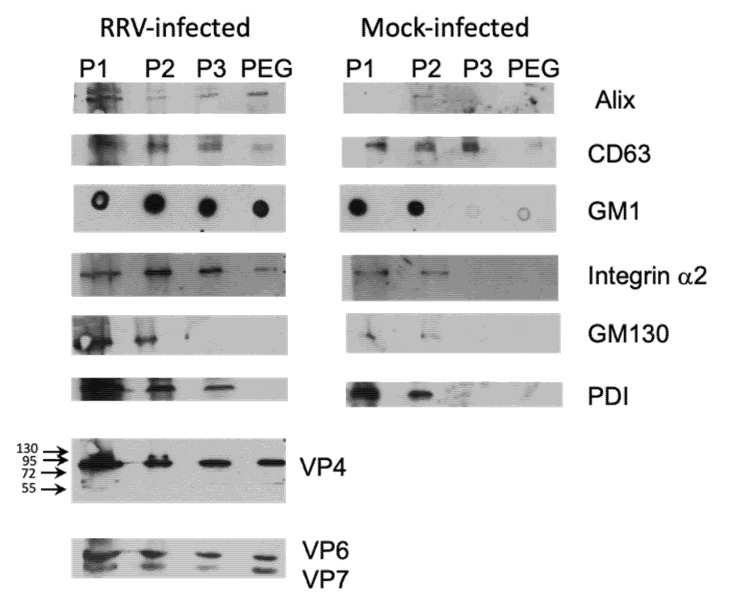
Presence of cellular markers and viral proteins in extracellular vesicles. Caco-2 cells were infected or mock-infected with strain RRV (MOI of 1) and at 18 hpi the supernatant was collected. Different fractions were obtained by differential centrifugation as described in material and methods; P1 at 500× *g*, P2 at 2000× *g*, P3 at 9600× *g* and PEG fraction after PEG6000 addition. All pelleted fractions were resuspended in 100 µL of TNC, and proteins from equal amounts of sample were pelleted by methanol/chloroform method, resuspended in Laemmli loading buffer, and separated in 10% SDS-PAGE gels. After separation, the proteins were transferred to nitrocellulose membrane and probed by specific monoclonal or polyclonal antibodies as described in material and methods. For detection of ganglioside GM1, 3 µL of sample were directly blotted onto nitrocellulose, air-dried, blocked by 5% milk in PBS and detected using cholera-toxin B subunit conjugated with biotin, followed by streptavidin peroxidase. Where needed, molecular size markers are indicated. Final detection was done using the Western Lightning Chemiluminescence Reagent Plus (Perkin Elmer Life Sciences, Boston, MA, USA).

Different cellular markers were tested to characterize the enriched EV. We compared EV obtained from infected and mock-infected Caco-2 cells, and observed that, in general, the fractions from infected cells contained a higher content of the proteins analyzed (Figure 4). P1 fraction, which probably contains complete detached cells presented high level of all proteins tested. Proteins known to localize in exosomes (ALIX and CD63) were present in different quantities in remaining fractions, with ALIX being present principally in PEG fraction, with lower amount being present in P2 and P3 fractions (Figure 4). Membrane markers GM1 and α2 integrin were tested to analyze the presence of microvesicles. Consequently, both these membrane markers showed high level in fractions P2 and P3, in which we could expect microvesicles of different sizes, and only small portion was present in PEG fraction (Figure 4). On the other hand, we evaluated the contamination of the fractions that should correspond to exosome, with intracellular organelles, and observed that the Golgi-specific protein GM130, and PDI, which localizes to the ER membrane, are absent in the PEG fraction. With respect to rotavirus proteins, they were present in all fractions derived from infected Caco-2 cells, and were absent in those obtained from mock-infected cells (Figure 4). We did not observe any important processing of the VP4 protein in the fractions analyzed, as detected by the VP5-specific monoclonal antibody HS2; VP4 (estimated size 88 kDa) was clearly visible, with a very low signal at 58 kDa (estimated size of VP5), suggesting that the VP4 protein of the viral particles associated with vesicles is not proteolytically cleaved and, therefore, the viral infectivity is not activated. These results suggest that method used is capable to separate, at least partially, different types of EV, and viral particles associated to these EV do not contain cleaved VP4 protein.

### 3.4. Particles Resembling Rotaviruses are Observed Associated with Extracellular Vesicles from the Outside and Inside

**Figure 5:**
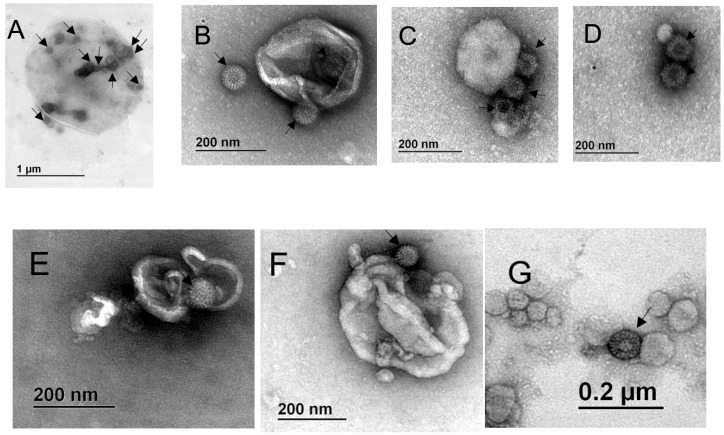
Rotavirus particles associate from the outside and inside of extracellular vesicles. Confluently grown MA104 and Caco-2 cells were infected with RRV strain, for 10 or 18 h, respectively, the time after which the cell culture supernatant was collected. Fraction P2 was obtained by centrifugation at 2000× *g*, P3 at 9600× *g* and PEG fraction by precipitation with PEG6000, and vesicles were then isolated using MagCapture^TM^ Exosome Isolation Kit PS. One drop of sample was fixed onto carbon vaporized coper grids and negatively stained with uranyl acetate. Samples were observed in EFTEM ZEISS Libra 120 electron microscope. (**A**) MA104 cells P2; (**B**) MA104 cells P3; (**C**,**D**) MA104 cells PEG; (**E**,**F**) Caco-2 cells P3; (**G**) Caco-2 cells PEG. Particles resembling rotavirus structure are pointed by arrows. Size bars are shown.

The observed increase in viral titer after treatment of the fractions with detergent suggested that a portion of the viral particles was present inside of the vesicles or, alternatively, that several viral particles are associated with single vesicles (from outside or inside) and their release results in an increased infectivity. To analyze the direct association of viral particles with vesicles, the EV present in the various fractions were further immuno-isolated using MagCapture^TM^ Exosome Isolation PS kit (Wako). The resulting EV were analyzed by transmission electron microscopy. Possible viral particles were identified by their characteristic wheel-like structure (pointed by arrows in Figure 5). We observed vesicles of different sizes, which apparently have viral particles associated from the outside, as well as present in the inside (Figure 5). In the case of fractions purified from MA104 cells we observed vesicles of about 1.5 µm and 350 nm, respectively in fractions P2 and P3, with several rotavirus resembling particles associated from the outside and present inside the EV (Figure 5A,B), while in the PEG fraction we observed smaller vesicles (40 to 200 nm) with several possibly viral particles associated from the outside (Figure 5C,D). In the case of fractions isolated from Caco-2 cells, we observed vesicles of about 300–400 nm, with what looked like viral particles being present inside and outside of the EV in the P3 fraction (Figure 5E,F), while the PEG fraction contained only smaller vesicles, with virus-like particles associated from the outside (Figure 5G).

### 3.5. Vesicle Associated Rotavirus Infectivity

**Figure 6:**
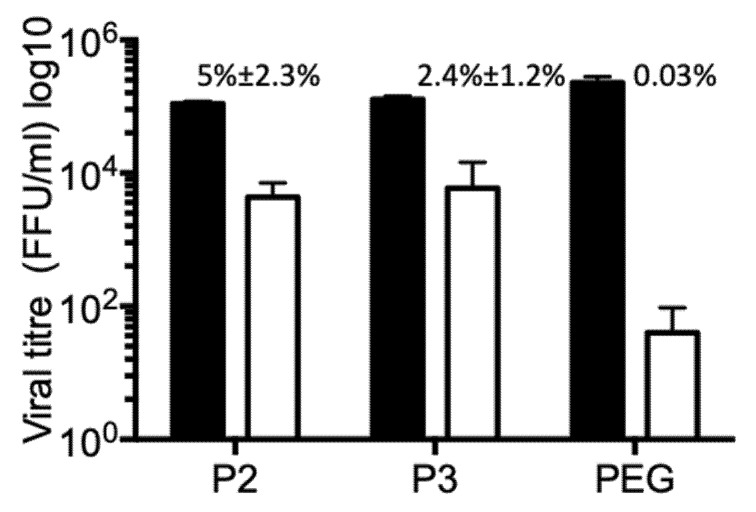
Viral particles associated with extracellular vesicles are protected from antibody neutralization. Caco-2 cells were infected with strain RRV (MOI of 1), and 18 hpi the supernatant was collected. Different fractions were obtained by differential centrifugation as described in material and methods; P2 at 2000× *g*, P3 at 9600× *g* and PEG fraction after PEG6000 addition. All pelleted fractions were resuspended in 100 µL of TNC, and an aliquot of the fraction was diluted 1/10 in MEM without serum. The diluted samples were divided into three equal aliquots, and subjected to incubation for 1 h with MEM (black bars), or neutralization with nMAb159 (open bars). Additionally, the membrane was solubilized by 0.1% Triton X-100 followed by neutralization by nMab159. After treatment with Triton X-100, the detergent was removed using HiPPR^TM^ Detergent Removal Columns. Then, the mixture was added to washed MA104 cells for 2 h to allow the virus cell entry, and the unbound vesicles were then removed by two rounds of washing. Infection was left to proceed for 15 h, and the infected cells were detected by immunostaining as described above. In fractions treated with detergent, followed by neutralization with nMab159, no infected cells were observed. The number above open bar represents percentage of infected cells after neutralization with nMAb159. Results represent the average of four independent experiments.

Rotavirus particles require trypsin treatment to enhance their infectivity. Without trypsin activation viral particles show some residual infectivity, approximately three logarithms lower than the infectivity of trypsin-activated virus. On the other hand, EV have intrinsic capacity to interact with membranes and enter cells. By this mechanism, they transport cytoplasmic cargo, mechanism by which cells seem to send signals to their surroundings informing about their physiological status. To test if rotaviruses are secreted from infected Caco-2 cells in association with EV, different vesicle fractions were incubated with MA104 cells. These EV fractions were not activated by trypsin treatment, to decrease the background of infection caused by possible free infectious viral particles. All tested fractions (P2, P3 and PEG) have certain capacity to infect permissive MA104 cells (Figure 6). To test if the viral particles were associated with EV from the outside, or they are present inside, the vesicles were incubated with nMAb159, to neutralize all possible free viral particles and also all viral particles attached to vesicle from the outside. It is clear that a portion of the viral particles present in fractions P2 and P3 were not neutralized by nMAb159, leaving 5% ± 2.3% (fraction P2) (n4), and 2.4% ± 1.2% fraction P3 (n4) of the original infectivity (Figure 6). In PEG fractions, which contained the highest number of infectious virions before nMAb159 neutralization, most of the infectivity was neutralized, suggesting that the majority of viral particles were accessible to nMAb159. It is important to mention that neutralization with nMAb159 after Triton X-100 membrane solubilization neutralized 100% infectivity in all fractions tested, confirming the high neutralizing capacity of this monoclonal antibody (results not shown). Observation that infectivity of some viral particles is refractory to action of nMAb159 confirms the possibility that portion of viral particles is present inside of some type of vesicle, possibly microvesicle.

## 4. Discussion

The paradigm of rotavirus cell release is changing in light of relatively recent data. Since it was originally proposed [10], it has been historically accepted that in non-polarized cells the new viral particles are released when the cells lyse as the consequence of virus replication. However, it has been recently reported that virus release occurs before cellular damage, in both polarized as well as in non-polarized cells [3,11,31]. An almost exclusive release of rotavirus particles before cell damage was also described in the human cholangiocyte cell line H69 [23], suggesting that this can be a general characteristic of this process. In this work, we confirmed earlier reports of pre-lysis release of viral particles from MA104 and also Caco-2 cells well before any damage to cell membrane is detected. In the case of MA104 cells, viral particles were found in the culture medium as early as 6 hpi, while in Caco-2 cells, infectious virus particles were observed at 12 hpi, the earliest time point analyzed. Previously, the secreted viral particles present in the supernatant obtained from infected MA104 cells were analyzed by density centrifugation, and they were found throughout the gradient, possibly associated with different types of membrane vesicles [31]. By differential centrifugation, we obtained a fraction (P3) that showed, by nanoparticles tracking analysis, it was composed of vesicles of heterogeneous sizes, ranging from 100 up to 450 nm. Therefore, it is possible that fraction P3 contains EV of distinct origins and characteristics, and deserve a further, more detailed analysis.

Given the heterogeneity of extracellular vesicles, we implemented a protocol composed of differential centrifugation, PEG6000 precipitation and affinity-isolation using a magnetic bead purification system to obtain vesicles of a more defined size. The comparison of secreted vesicles derived from infected and mock-infected cells showed that rotavirus infection stimulates their secretion, given that proteins and vesicles analyzed by SDS-PAGE or microfluid tracking show a large increase after infection. A similar increase in the quantity of proteins associated with vesicles after rotavirus infection was observed previously [22]. Several other viruses have been also described to increase vesicle secretion after infection; among them, herpes simplex virus 1 (HSV-1) [20,33], tick-borne Langat virus [34], and human immunodeficiency virus HIV [35,36], while hepatitis C virus (HCV) does not seem to affect vesicle secretion [13]. Increased vesicle secretion could be used on one hand by viruses to disseminate in association with these EV; on the other hand, cells could disseminate signals to protect surrounding cells from infection.

By transmission electron microscopy analysis of the affinity purified EV, we observed possible rotavirus particles associated with large vesicles (possibly microvesicles, originated from the plasma membrane) as well as with smaller vesicles (possibly exosomes). Some of the viral particles were present inside the vesicles, but most of them were associated from the outside, especially with vesicles that correspond in size to exosomes. The association of viral particles with the outside of EV, although not very common, has been described previously for several viruses, like polyomavirus [14], HSV-1 [33], and adenovirus [18]. It is not clear how the association of viral particles with vesicles from outside occurs, if it is a result of interaction during cell exit, or if this interaction occurs after viral particles and EV can interact outside of the cells. Possible roles of this interaction during virus cell cycle are also not clear, and more studies should be directed to clarify this point. In the case of HIV, it was described that the viral particles could be entrapped in aggregates of exosomes which, apart from protecting the virus from the immunological system seem to increase viral infection and replication [37]. The external interaction between viral particles and extracellular vesicles is reminiscent of the recently discovered interactions between bacteria and some enteric viruses, where bacteria seem to increase viral fitness, infection, and recombination [38,39,40]. In these cases, it has been suggested that bacteria serve as concentrators of virus particles, increasing the MOI. It is not clear at what point of the replication cycle rotavirus particles associate with secreted vesicles, although given the large amount of free viral particles secreted, it is possible that external association with EV could occur outside of the cells, after vesicle secretion. It remains to be investigated if these externally associated viral particles acquire some new biological qualities.

The viral particles present inside the vesicles should, in principle, be protected from extracellular factors, like antibodies. In agreement with this, we observed that a fraction of the viral particles associated with larger vesicles, in which they seem to be present inside, was protected from neutralization with the highly efficient nMab159, while almost no protection was observed in viruses associated with vesicles in the PEG-purified fraction. Different viruses have been shown to be present inside secreted extracellular vesicles, and as consequence to be inaccessible to neutralization by monoclonal antibodies, and to acquire the ability to infect cells which lack the corresponding viral receptor. For example, the association of viral particles with vesicles protected from antibody neutralization hepatitis E virus [15], and HCV [13,41], while allowed the receptor independent infection of adenovirus and bunyavirus [16,18]. Additionally, the JC polyomavirus particles associated with vesicles were resistant to treatment of cells with a receptor destroying enzyme, and could enter cells through a receptor-independent pathway, suggesting a role of vesicles in the dissemination and spread of JC polyomavirus in the central nervous system [14]. Similarly, HSV-1 associated to vesicles was able to infect CHO cells (which are refractory to infection with free HSV-1 particles), and this infection was resistant to neutralization with anti-HSV-1 antibodies [33]. Additionally, in case of HCV, the transmission of infection to permissive cells by association of replication-competent subgenomic RNA with exosomes has been observed [42], opening an additional possibility for a novel mechanism of viral spread and immune escape.

In the case of rotavirus, Santiana et al. observed the secretion of viruses, from human cholangiocytes-infected H69 cells, inside large vesicles, and similar vesicles were also found in stools of experimentally infected pigs and mice [23]; no viral particles associated to the outside of the vesicles were observed. Interestingly, viral particles associated with these large vesicles purified from stools or from H69 cells showed a proteolytically-cleaved VP4 protein, while viral particles purified from MA104 and Caco-2 cells contained uncleaved VP4 [23], suggesting that protease cleavage was cell line specific. In accordance with these results, we did not observe VP4 processing in the viral particles associated with the purified EV. Additionally, viral particles secreted inside vesicles purified from H69 cells showed enhanced infectivity and disease severity, as compared to individual viral particles [23], while we did not observe a higher infectivity associated with vesicle-bound viral particles when compared to free viruses. Despite the lack of VP4 processing, we observed certain level of infectivity in the vesicle-associated viral particles, which remained even after neutralization with a potent neutralizing monoclonal antibody directed to VP7. Protection from neutralization suggests that at least part of the viral particles are present inside vesicles, thus protected from the activity of neutralizing antibodies. These observations also suggest the possibility that rotavirus does not need to have a trypsin-cleaved VP4 to be infectious if it is introduced to the cytoplasm by a receptor-independent mechanism; for example, association with vesicles.

For a long time, rotavirus infection has been thought to be limited to the mature enterocytes in the small intestine. However, in the last decade several reports have described the presence of rotavirus outside the intestinal lumen. Thus, rotavirus antigens, and even infectious particles, have been found in the blood of both animal models and humans after infection, even in the absence of clinical symptoms (diarrhea) [43,44,45]. Rotavirus antigen or dsRNA were also found in other organs outside the intestine, specifically spleen, kidney, liver, lung, heart or testes [46,47]. Additionally, rotavirus has also been associated with direct infection of the central nervous system, and it was found in spinal fluid of humans or in experimental animal models [48,49]. In general, it appears that the rotavirus-infected host will present at least a short period of viremia, and the virus could be detected in other organs, even in immunocompetent individuals. However, the mechanism of extraintestinal spread and dissemination throughout the body is not understood. The description of the association of rotavirus particles with EV suggests the possibility that the virus may enter different types of cells in a receptor-independent manner. In this regard, the association of viral particles with extracellular vesicles has been reported to permit different viral species to infect cells, disseminating to organs previously not thought to be infected. Given that rotavirus dsRNA, antigen or infectious virus particles are readily found in the serum of hosts with rotavirus infection, opens up the possibility that at least some of these particles could be associated with EV (externally or internally), and could be disseminated throughout the body.

Previously it has been shown that rotaviruses interact with detergent resistant domains, rafts, during their cell exit [12], and it was shown that rotavirus-raft interaction is mediated by VP4, which is specifically targeted to these domains [50], and by NSP4 protein, the DLP receptor in the endoplasmic reticulum [8,50,51]. In light of the recent description of interactions of rotavirus particles with EV, it is of interest that the above mentioned detergent-resistant domains (rafts) have also been implicated in the release of extracellular vesicles, and ceramides (building blocks of rafts) are important in formation of these vesicles [52,53]. It has been even proposed that exosomes could be responsible for exchange of rafts between cells [52]. Further studies should be focused on characterization of these interactions.

## 5. Conclusions

Despite years of intensive research into rotavirus cell cycle, the viral release from infected cells is still not well understood. In this work, we confirm non-lytic release of viral particles from two cell lines. The viral infection stimulated secretion of different types of extracellular vesicles. Particles which, by their morphology, resemble rotavirus were observed associated with vesicles, some being present inside but also bound from outside. Protein immunodetection, sedimentation characteristics and direct electron microscopy observation suggest that vesicles which contain viral particles inside could be microvesicles, shielding them from external environment. These vesicles were able to promote infection of viral particles without trypsin activation. Furthermore, we observed that portion of viral particles (probably ones present inside the vesicles), were refractory to neutralization with potent neutralizing monoclonal antibody. All these results point to importance of extracellular vesicles in rotavirus infection.
